# Revising on the run or studying on the sofa: prospective associations between physical activity, sedentary behaviour, and exam results in British adolescents

**DOI:** 10.1186/s12966-015-0269-2

**Published:** 2015-09-04

**Authors:** Kirsten Corder, Andrew J. Atkin, Diane J. Bamber, Soren Brage, Valerie J. Dunn, Ulf Ekelund, Matthew Owens, Esther M. F. van Sluijs, Ian M. Goodyer

**Affiliations:** MRC Epidemiology Unit, University of Cambridge School of Clinical Medicine, Box 285 Institute of Metabolic Science, Cambridge Biomedical Campus, Cambridge, CB2 0QQ UK; UKCRC Centre for Diet and Activity Research (CEDAR), MRC Epidemiology Unit, University of Cambridge School of Clinical Medicine, Box 285 Institute of Metabolic Science, Cambridge Biomedical Campus, Cambridge, CB2 0QQ UK; Developmental Psychiatry Section, Department of Psychiatry, University of Cambridge, Douglas House, 18b Trumpington Road, Cambridge, CB2 8AH UK; Cambridge and Peterborough NHS Foundation Trust, Cambridge, UK; Department of Sports Medicine, Norwegian School of Sports Sciences, Oslo, Norway; Mood Disorders Centre, University of Exeter, Perry Road, Exeter, EX4 4QG UK

**Keywords:** Physical activity, Academic performance, Adolescent, Sedentary behaviour, Television viewing

## Abstract

**Background:**

We investigated prospective associations between physical activity/sedentary behaviour (PA/SED) and General Certificate of Secondary Education (GCSE) results in British adolescents.

**Methods:**

Exposures were objective PA/SED and self-reported sedentary behaviours (screen (TV, Internet, Computer Games)/non-screen (homework, reading)) measured in 845 adolescents (14·5y ± 0·5y; 43·6 % male). GCSE results at 16y were obtained from national records. Associations between exposures and academic performance (total exam points) were assessed using multilevel mixed-effects linear regression adjusted for mood, BMI z-score, deprivation, sex, season and school; potential interactions were investigated.

**Results:**

PA was not associated with academic performance. One-hour more accelerometer-assessed SED was associated with (β(95 % CI)) 6·9(1·5,12·4) more GCSE points. An extra hour of screen time was associated with 9.3(−14·3,-4·3) fewer points whereas an extra hour of non-screen time (reading/homework) was associated with 23·1(14·6,31·6) more points. Screen time was still associated with poorer scores after adjusting for objective PA/SED and reading/homework.

**Conclusions:**

An extra hour/day of screen time at 14·5y is approximately equivalent to two fewer GCSE grades (e.g., from B to D) at 16y. Strategies to achieve the right balance between screen and non-screen time may be important for improving academic performance. Concerns that encouraging more physical activity may result in decreased academic performance seem unfounded.

## Background

Physical inactivity is associated with increased risk of obesity and related metabolic disorders among adolescents [1]. Physical activity has physiological benefits and is positively associated with mental health and enhancement of brain function and cognition [2, 3]. It has been hypothesised that physical activity may enhance academic performance among young people [4–6].

Sedentary behaviours are distinct from a lack of physical activity, such that both TV viewing and too little moderate to vigorous physical activity (MVPA) may be independently detrimental to health [7]. A recent systematic review concluded that screen based behaviours are longitudinally associated with obesity independent from subcomponents of physical activity in young people [8]. A lot of sedentary time, specifically TV viewing, could also be detrimental to cognitive development [9].

A systematic review based on 14 studies concluded a positive relationship between baseline physical activity and future school performance [4]. Due to methodological issues regarding the review and the individual studies, the validity of this conclusion has been questioned [10]. Other recent reviews also conclude overall positive associations between academic performance and physical activity but question the quality of evidence, including the lack of prospective and objective studies [11, 12]. Evidence is also limited by the use of self-reported academic markers, which are susceptible to recall bias. Recent research has indicated the possibility of a non-linear association between physical activity and academic performance [13, 14] although this has rarely been examined. Screen behaviours have differing associations with academic performance [15–18]. TV viewing has been shown to have either a negative association or no association with academic performance; [9, 17] results for internet use are also equivocal [15, 16]. Among Finnish children, there was no association between objectively measured sedentary behaviour and academic performance, and a negative association between combined screen behaviours and academic performance [18]. A British study showed positive associations between percentage time in MVPA at 11y with boys’ academic performance in English at 13y and 16y, and with English at 13y and Science at 16y among girls [19]. These associations were examined for English, Mathematics and Science, not overall academic performance, and sedentary behaviour was not examined. Studying the association between activity-related behaviours and academic performance could have important implications as school sports appear to have been reduced to give preference to academic subjects [20].

General Certificate of Secondary Education (GCSE) examinations taken at 16y (Year 11) at the end of compulsory education in England, Wales and Northern Ireland are used nationwide to compare academic performance. We investigated the association between objectively measured physical activity at 14 · 5y (Year 10) and GCSE results at 16y (Year 11). We hypothesised that objective physical activity and self-reported non-screen sedentary behaviour would be positively associated, and objective sedentary behaviour, and self-reported screen time negatively associated with GCSE results.

## Methods

Participants were 845 adolescents (43.6 % male, Mean ± SD 14·5 ± 0·5y) from the ROOTS study [21]. ROOTS is a prospective cohort aiming to determine relative contributions of genetic, physiological, psychological and social variables to well-being and mental health during adolescence.

Secondary schools from Cambridgeshire and Suffolk, UK, ranging 30 miles north, 20 miles south and 20 miles west of Cambridge were approached. Of 27 schools invited, 18 agreed to participate. Study information, invitation letters and parent/student consent forms were sent to eligible parents and students via schools; 1238 adolescents completing these forms at age 14 were invited into the study. A second information sheet and consent form regarding physical activity assessments were sent to baseline participants; those returning completed consent forms were invited to measurements at their school. The current study used data from the first physical activity assessment, approximately six months after baseline (November 2005 to July 2007). ROOTS was approved by the Cambridge local research ethics committee (Cambridge University Research Ethics Committee) (reference 03/302).

Trained researchers administered questionnaires, conducted physical measurements and gave instructions regarding physical activity measurements at participating schools. Height was measured to the nearest 0·1 cm (Leicester Height Meter, Invicta Plastics, Leicester, UK), weight was measured to the nearest 0·1 kg (Tanita TBF-300 MA, Tanita, Tokyo, Japan) in light clothing without shoes and socks. Height and weight were used to calculate body mass index (BMI, kg/m^2^). BMI z-score and weight status were derived using sex- and age-dependent cut-points [22].

### Academic performance

GCSEs are examinations taken at the end of Year 11 (16y) by the majority students at the end of compulsory education in England, Wales and Northern Ireland. In most schools at the time it was compulsory to take GCSEs in English, Maths, Sciences, and a foreign language with a choice of other GCSEs in subjects such as History and Geography. Students usually study for two years before completing GCSE examinations and each student takes eight or nine GCSEs as standard, with flexibility depending on student ability and school policy.

GCSE results were obtained from the Department for Education National Pupil Database. Academic performance was calculated as the sum of grade based points (A* = 58, A = 52…G = 16) as per the national reporting standard [23].

### Objective sedentary and physical activity measurement

Objective sedentary time and physical activity were assessed using combined heart rate and movement sensing (Actiheart, CamNtech, Papworth, UK) [24–26]. The monitor clips onto two ECG electrodes and was positioned in the midline, just below the xiphisternum and attached via a 70-100 mm wire to a smaller clip, horizontally to the left chest wall. The data was collected in 30s epochs. Participants were instructed to wear the monitor continuously for the remainder of the testing day and for four consecutive days, including two weekend days.

Full details for physical activity processing are available elsewhere [27]. Briefly, heart rate data were individually calibrated [28], and combined with acceleration using branched equation modelling [29], which compares favourably with indirect calorimetry [24, 25]. Self-reported sleep times were overlaid on objective data and visually inspected to derive time spent awake or asleep. Only participants who provided ≥48 h of monitor data were included in analyses. MVPA was defined as time ≥4 standard metabolic equivalents (METs) as described previously [27]. Sedentary time was defined as non-sleep activity at <1·5 METs [30]. Season of physical activity measurement was derived using the date of measurement, classified as Winter (December-February), Spring (March-May), Summer (June-August) and Autumn (September-November).

### Self-reported sedentary time

Separately for week and weekend days, adolescents reported daily time spent in the following sedentary behaviours: watching TV (including video/DVD), using the internet, playing video games, doing homework, and reading for pleasure. A weighted mean ((5*weekday + 2*weekend)/7) of daily time spent in each behaviour was calculated. These were summed into time spent in screen behaviours (TV, internet, computer games) and non-screen behaviours (reading, homework). The question used did not allow differentiation between screen and non-screen homework so all homework reported was classed as non-screen for this analysis. Although this data has been used previously [31], the reliability and validity of this instrument has not been assessed.

### Moods and feelings

The Mood and Feelings Questionnaire (MFQ) is a 33-item self-report measure of depressed mood over the prior two weeks [32]. The MFQ was administered at 14y, six months before physical activity data collection and has validity as a screen for adolescents with unipolar depression [32]. Respondents are scored on a 3-point scale (mostly/sometimes/never); higher MFQ scores indicate increased risk for unipolar depression with current levels >25 indicating the clinical range.^26,27^ Internal consistency in this sample was high (Cronbach's alpha = 0·96).

### Socio-economic status

The Index of Multiple Deprivation (IMD) was computed using home postcode at ROOTS baseline. The IMD is used for ranking area-level deprivation in England [33]. This measure combines information on deprivation from 37 domains including income, employment, health, and crime which are weighted and combined. The IMD is based on Lower Super Output Areas (LSOA) with an average of 1500 people living in each. The LSOA ranked one is the most deprived and 32,482 is the least deprived; the range within ROOTS was 1199 to 32,319. We use IMD 2007 which represents the situation in 2005. The detailed derivation of this measure is described elsewhere [33].

### Statistics

Due to skewness, data for most sedentary/physical activity variables, deprivation and mood are presented as median and inter-quartile range (IQR) but as these were exposure variables, they were not transformed for analysis. The distribution of residuals was checked to make sure that this was appropriate.

Sex differences in descriptive characteristics, academic performance and sedentary/physical activity variables were assessed using Students t-tests, Wilcoxon-Mann–Whitney tests or Chi-square tests as appropriate.

Associations between exposure variables (MVPA, sedentary time, combined screen time and non-screen time) at 14·5y and academic performance at 16y were investigated using multilevel linear regression. All models were adjusted for mood, BMI z-score, deprivation, sex, season of measurement and nesting of participants within schools. Associations were first tested with each exposure variable separately before all exposure variables were put into the same model to test for independence of associations.

Interactions between sedentary/physical activity variables and sex, IMD, and BMI z-score were investigated in further models which included only one sedentary/physical activity exposure variable.

Due to previous research showing non-linear associations (inversed u-shape distribution) between physical activity and academic performance [34], we added a quadratic term for each exposure to test this.

Sensitivity analyses were conducted using points per qualification to represent academic performance in models including one sedentary/physical activity exposure variable (data not shown).

## Results

Of 1238 adolescents (97 % of cohort) invited, 998 (83 %) adolescents and parents completed postal informed consent and 931 (93 % of those consenting) attended measurements. Compared to baseline participants, those with missing sedentary/physical activity data did not significantly differ in their academic performance (*p* = 0·58), sex (*p* = 0·07) or BMI z-score (*p* = 0·92) but participants had worse current mood scores (*p* = 0·01) and were less deprived (*p* = 0·01). Data from 845 adolescents (84·7 % of those consenting and 90·8 % of those attending measurements) with complete academic performance and sedentary/physical activity data were included in analyses.

Descriptive characteristics are summarised (Table 1). Boys were more active and less sedentary than girls; boys reported more screen time, but less non-screen sedentary time than girls. Girls had higher academic performance than boys.

**Table 1:** Participant characteristics. Values are Mean(SD) unless otherwise stated

	All	Boys	Girls	*p* value for difference
	(n = 845)	(n = 368)	(n = 477)	
Demographic variables				
Age	14·5 (0·5)	14·5 (0·5)	14·6 (0·5)	0·04
BMI z-score	0·20 (1·06)	0·19 (1·08)	0·20 (1·04)	0·83
Deprivation rank	24,455 (1199, 32319)	24,4890 (6098, 32319)	24,455 (1199, 32319)	0·20^a^
MFQ	13 (8–20)	11 (7–17)	15 (8–23)	<0·001^a^
Objective sedentary (mean hr/day)				
Sedentary	6·1 (2·1)	5·6 (2·0)	6·5 (2·1)	<0·001
Objective physical activity (median (IQR) min/day)				
MVPA	38·8 (19·7-65·1)	56·4 (31·2- 82·9)	28·0 (14·0- 48·6)	<0·001^a^
Self-reported sedentary (median (IQR) hr/day)				
*Combined variables*				
Screen time	4·0 (2·8-5·5)	4·3 (3·1-5·7)	3·7 (2·6-5·1)	<0·001^a^
Non screen sedentary	1·5 (1·0-2·3)	1·3 (0·7-2·0)	1·6 (1·1-2·6)	<0·001^a^
*Individual variables*				
TV viewing	1·9 (1·2-2·7)	1·9 (1·3-2·9)	1·9 (1·1-2·7)	0·436^a^
Internet Use	1·3 (0·6-2·3)	1·1 (0·5-2·0)	1·6 (0·9-2·4)	<0·001^a^
Computer Games	0·1 (0·0-0·9)	0·9 (0·3-1·6)	0·0 (0·0-0·1)	<0·001^a^
Homework	1·0 (0·6-1·6)	1·0 (0·6-1·3)	1·2 (0·8-1·8)	<0·001^a^
Reading	0·3 (0·0-0·8)	0·2 (0·0-0·6)	0·5 (0·0-0·8)	<0·001^a^
Academic achievement				
Total points^1^	416·6 (117·5)	394·1 (115·1)	434·0 (116·6)	<0·001

^a^Wilcoxon-Mann–Whitney test

^1^Calculated to include GCSE and GNVQ results e.g., for GCSEs A* = 58, A = 52…G = 16, and similar for GNVQ grades

**Fig. 1:**
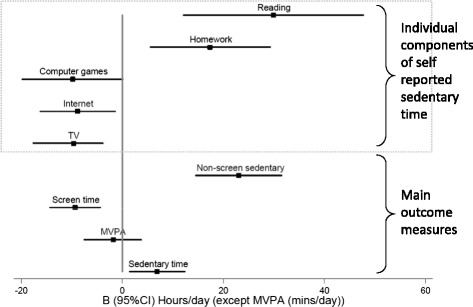
Results are from two level linear regression models adjusted for MFQ, BMI z-score, deprivation, season of physical activity measurement and sex. Results are shown for objectively measured sedentary time and moderate and vigorous physical activity (MVPA), and self-reported separate and composite screen time (TV, Internet and Computer Games) and non-screen sedentary behaviours (homework and reading). Values represent adjusted associations (Beta (95 % CI)) between academic achievement (total GCSE points) and sedentary variables (hours/day) and MVPA (mins/day)

Results showing associations between all exposures and academic performance are shown in Table 2 and also displayed in Fig. 1. MVPA was not significantly associated with academic performance. Objectively measured sedentary time was positively associated with academic performance. Differential effects by type of sedentary behaviour were observed. Screen time was associated with fewer total GCSE points but conversely, non-screen sedentary time (reading/homework) was associated with higher academic performance. Self-reported sedentary variables remained significant when mutually adjusted and when adjusted for objective sedentary time and MVPA indicating independent associations with academic performance.

**Table 2:** Adjusted associations between academic achievement and physical activity/sedentary variables from multiple multilevel linear regression models

		Total points^a^	
		Β (95 % CI)	*P* value
In separate models			
Sedentary time	(hr/day)	6·9 (1·5, 12·4)	0·016
MVPA	(min/day)	−1·8 (−7·5, 3·8)	0·497
Screen time	(hr/day)	−9·3 (−14·3, −4·3)	0·001
Non-screen sedentary	(hr/day)	23·1 (14·6, 31·6)	<0·001
Mutually adjusted			
Sedentary time	(hr/day)	6·2 (0·6, 11·9)	0·033
MVPA	(min/day)	1·0 (−5·7, 5·8)	0·996
Screen time	(hr/day)	−9·1 (−14·5, −3·7)	0·003
Non-screen sedentary	(hr/day)	24·7 (17·3, 32·0)	<0·001
Quadratic functions			
Non-screen sedentary quadratic	(hr/day)^2	−8·3 (−12·3, −4·3)	<0·001
Non-screen sedentary	(hr/day)	67·6 (44·8, 90·3)	<0·001

All models clustered for school and adjusted for MFQ, BMI z-score, deprivation, season of physical activity measurement, and sex

^a^Calculated to include GCSE and GNVQ results e.g., for GCSEs A* = 58, A = 52…G = 16, and similar for GNVQ grades

Sedentary hr/day spent <1.5 METS

MVPA mean mins/day spent in moderate and vigorous physical activity above 4 METS

Screen time sedentary includes self-reported time spent in TV/DVD, Internet and Computer games

Non Screen time sedentary includes self-reported time spent in homework and reading

Quadratic functions are only shown if significant

There were no significant interactions between exposure variables and sex (all *p* > 0.547). There was an interaction between MVPA and BMI z-score (β(95 % CI)) 3·7(0·1, 7·3) *p* = 0.046), but in stratified analyses neither approached significance but the association was negative for those with lower BMI (−3.9 (−10.7,2.9) *p* = 0.246) and positive for those with higher BMI (1.2 (−3.9,6.4) *p* = 0.616).

The only quadratic association was for non-screen sedentary time and academic performance (Table 2). After graphical examination, this suggests an inverted ‘u’ shaped association (peaking around 4 h/day); wherein a stronger positive association was observed in the middle of the non-screen spectrum with those who have lowest and highest non-screen sedentary time having lower academic performance.

*Post hoc* analyses exploring the associations with specific types of sedentary behaviours (Table 3 and Fig. 1) showed that TV viewing and Internet use were separately negatively associated with academic performance whereas all non-screen behaviours were positively associated with academic performance.

**Table 3:** Adjusted associations between academic achievement and separate screen and non-screen sedentary variables in multiple multilevel linear regression models

		Total points^a^	
		Β (95 % CI)	*P* value
Screen behaviours (in the same model)			
TV viewing	(hr/day)	−9·6 (−17·6, −3·8)	0·011
Internet use	(hr/day)	−8·8 (−16·2, −1·4)	0·023
Computer games	(hr/day)	−9·8 (−19·8, 0·1)	0·052
Non-screen behaviours (in the same model)			
Homework	(hr/day)	17·4 (5·6, 29·3)	0·007
Reading	(hr/day)	29·9 (12·1, 47·7)	0·003

All models clustered for school and adjusted for MFQ, BMI z-score, deprivation, season of physical activity measurement, and sex

^a^Calculated to include GCSE and GNVQ results e.g., for GCSEs A* = 58, A = 52…G = 16, and similar for GNVQ grades

Results from sensitivity analyses using points per qualification to represent academic performance were not meaningfully different to those presented here (results not shown).

## Discussion

Adolescents reporting more screen time at 14·5y had lower GCSE results at 16y. An extra hour of daily screen time at 14·5y predicted 9.3 fewer GCSE points at 16y (equivalent to two grades lower such as from a B to a D). However, participants doing an extra hour of daily homework and reading (up to 4 h/day) predicted 23.1 more GCSE points (equivalent to one whole GCSE (e.g., Grade F = 22 points)). These associations are independent suggesting that irrespective of the amount of reading and homework, screen time is still detrimental to GCSE performance, to the extent of 9 points or half a GCSE (at Grade G).

Contrary to our hypothesis, but similar to some recent findings [18], there was no association between academic performance and MVPA. Our findings are contrary to a recent British study which concluded a positive association between MVPA at 11y and GCSE results (at 16y) in English for boys and Science for girls [19]. These associations differed by subject and as we used a composite measure of all GCSEs taken [19], this may explain the differences. The previous study adjusted analyses for overall activity (counts per minute) which had a negative association with academic performance in their unadjusted analyses [19]; this highlights the complex nature of this association and the sensitivity of results to the analysis strategy and variables used. Recent systematic reviews have concluded a positive association between academic performance and physical activity [4, 11, 12]. The reviews highlight the variation in adjustment for a range of covariates, most commonly sex and ethnicity, and also call for further considering mental health, socio-economic status and BMI [12]. Differences could also be due to measurement methods, population differences, educational systems or a combination of the above factors. Objectively measured data usually provides a more ‘valid’ estimate of activity duration and intensity than self-report data but it is unable to assess time spent in specific behaviours. The lack of activity-dependent information may mask the identification of associations such as those between screen and non-screen sedentary time shown here. Results show that engaging in physical activity is not detrimental to academic performance. The benefits of physical activity for health should still put physical activity promotion as public health priority.

Higher screen use at 14·5y led to lower GCSE grades at 16y, similar to that shown in younger Finnish children [18]. An extra hour/day of screen time led to approximately 9 less GCSE points. This is especially important when considering that the median daily screen time was approximately 1·9 h; 2 h/day of screen use equates to 18 fewer points, equivalent to 4 grades (e.g., between A* and C) or approximately a whole GCSE (Grade G = 16 points). This appears to support the recommendation of <1 to 2 h/day of entertainment screen time for children [35]. All three separate screen behaviours were independently negatively associated with academic performance. This suggests that parents concerned about their child’s academic performance could consider limiting TV time, and also internet use and computer games. There is prior evidence that doing more homework is associated with higher academic performance,^30^ however, results from *post hoc* analyses suggested that both reading and homework were independently associated with academic performance.

Our results indicate that objectively measured sedentary time is positively associated with academic performance whereas our self-report behavioural items show a negative association as hypothesised. It is possible that the differing associations observed between screen time and non-screen time could explain why objectively measured sedentary time was not strongly associated with academic performance. As both screen and non-screen time would be captured by the objective estimate, the opposite directions of their associations may cancel each other out. Further, the objective measure will encompass many more types of sedentary behaviour throughout the day which may also have differential associations with academic performance. Without more detailed time use data, we are unable to further investigate what other behaviours are being conducted that could explain this result.

There appears to be an inverse ‘u’ shaped relationship between non-screen sedentary behaviour and academic performance, with the association peaking at 4 h/day; participants doing the least and most reading and homework had lower academic performance. As only 52 participants reported >4 h of non-screen time this association appears more relevant at the lower end of the distribution with homework/reading up to 4 h/day appearing to be beneficial for academic performance. We could hypothesize that the extreme ends of this inverse ‘u’ shaped distribution include participants who do little homework and achieve low exam results and also participants who are struggling at school, and therefore do a lot of homework, but unfortunately also performed badly in exams. It is also possible that the amount of reading and homework set is likely to differ depending on what subjects are being studied. It is possible that the less academic students are likely to be doing the less academic subjects and may be set less homework. Screen and non-screen sedentary time at 14·5y had independent associations with subsequent academic performance suggesting that even if participants do a lot of reading and homework, screen time is still detrimental to later academic performance.

To our knowledge, this is the first time that prospective associations between GCSE results and sedentary behaviours have been examined. This is relevant to the vast majority of adolescents from England, Wales and Northern Ireland as GCSEs are still currently used in the UK. Missing data was more likely among those with higher mood scores and those who were slightly less deprived so this may also limit external validity of our results. Current mood was measured six months before physical activity and the vast majority of participants had low to moderate scores outside the clinical range. Students would have been approximately six months into their GCSE courses during the sedentary/physical activity measurements so this is relevant to the exams taken but it is possible that sedentary behaviour and physical activity may have changed over the subsequent months. The lack of control for exposures measured at 16y is a limitation; a measure of attainment or other relevant measure such as IQ or cognition, taken from the same time as the exposure would have allowed stronger conclusions to be drawn. All homework reported was classed as non-screen time and it is possible that screen-based homework was done which may have influenced our results. Further, it is likely that rapidly developing technology and increased availability of screen-based media since the study was conducted has contributed to an altered profile of adolescent sedentary behaviour over time. The instrument used to assess self-reported sedentary behaviour has not been validated and our results should be interpreted accordingly. We examined whole day physical activity so that our results are relevant to the British physical activity guidelines of 60 min each day; however, it is possible that the inclusion of predominantly sedentary school time may be diluting associations. Our analyses are prospective therefore allowing cautious inferences about direction of association; however it would be impossible to tell whether reductions in screen time caused an increase in academic performance without a randomised controlled trial.

## Conclusions

Screen time was associated with lower academic performance, suggesting that strategies to limit screen behaviours among adolescents may benefit academic performance. Homework and reading were positively associated with GCSE results and can be seen as legitimate targets for policy makers and educationalists in the early teenage years. Findings suggest an optimal policy is required whereby neither low nor excessive practices are encouraged. Implementation of greater priority by parents to reduce screen based behaviours involving TV, Internet or Computer Games, are also suggested. Physical activity does not appear to be either detrimental or beneficial to academic performance, suggesting that emphasis on physical activity promotion for health benefits might be most appropriate.

## Abbreviations

BMI: Body mass index
cm: centimetre
DVD: Digital versatile disc
GCSE: General Certificate of Secondary Education
hr/d: Hours per day
IMD: Index of multiple deprivation
IQR: Inter-quartile range
kg: Kilogram
kg/m^2^: Kilogram per square metre
LSOA: Lower super output areas
METs: Standard metabolic equivalents
MFQ: Mood and feelings questionnaire
mm: Millimetre
MVPA: Moderate and vigorous physical activity
PA: Physical activity
ROOTS: Study name (not an acronym)
s: Seconds
SED: Objectively measured sedentary time
TV: Television
UK: United Kingdom
y: Years
